# Emergence of Exhausted B Cells in Asymptomatic HIV-1-Infected Patients Naïve for HAART is Related to Reduced Immune Surveillance

**DOI:** 10.1155/2012/829584

**Published:** 2012-03-05

**Authors:** Manuela Fogli, Carlo Torti, Fabio Malacarne, Simona Fiorentini, Melania Albani, Ilaria Izzo, Cinzia Giagulli, Fabrizio Maggi, Giampiero Carosi, Arnaldo Caruso

**Affiliations:** ^1^Section of Microbiology, Department of Experimental and Applied Medicine, University of Brescia, Spedali Civili Square, 25123 Brescia, Italy; ^2^Institute of Infectious Tropical Diseases, School of Medicine, University of Brescia, Spedali Civili Square, 25123 Brescia, Italy; ^3^Virology Section and Retrovirus Centre, Department of Experimental Pathology, University of Pisa, 35-37 San Zeno Road, 56127 Pisa, Italy

## Abstract

Alterations of B cell subpopulations have been described up to date as characterizing advanced stage of HIV-1 infection. However, whether such defects are relevant in subjects with a preserved number of CD4^+^ T cells (>350 cells/*μ*L) is unclear. In a cross-sectional study, we investigated if signs of B cells exhaustion and impaired viral immune surveillance are present in a cohort of 43 asymptomatic HIV-1-infected patients with preserved CD4^+^ T cell counts (>350 cells/*μ*L) and highly active antiretroviral therapy (HAART) untreated. A dramatic expansion of exhausted tissue-like memory B cells (CD10^−^CD21^low^CD27^−^) was observed. B cells alteration was related to an increase in Torque teno virus (TTV) load, used as surrogate marker of immune function. Successfully HAART-treated patients showed normalization of B cell subpopulations frequency and TTV load. These results provide new insights on B cell in HIV-1 infection and show that development of B cell abnormalities precedes CD4^+^ T cell decline.

## 1. Introduction

Through direct or indirect effects, HIV-1 infection leads to several perturbations on most cells of the immune system. The persistent viral replication induces loss of CD4^+^ T cells and a general chronic cellular activation that affect viability, subsets distribution, phenotype, and function of all the major immune cell populations [1]. B cells exhibit numerous effects of HIV-1-induced immune activation. Increased expression of activation and proliferation markers, polyclonal B cell hyperactivation, and hypergammaglobulinemia are some of the B cell defects detectable in HIV-1-infected patients [2, 3]. Moreover, B cells derived from HIV-1-infected patients show several alterations *in vitro*, as high spontaneous antibodies secretion, defective responses to B cell stimuli, and impaired costimulatory functions [4–8]. These functional defects have been recently linked to perturbation in the frequency of several B cell subpopulations. Naïve B cells (CD21^high^CD27^−^) constitute the largest B cell population in the blood of healthy individuals, followed by memory B cells (CD21^high^CD27^+^). The frequency of memory B cells is strongly decreased in HIV-1-infected individuals, and this occurrence is accompanied by an increased levels of naïve B cells, activated mature B cells (CD20^+^CD21^low^CD27^+^), and plasma cells (CD20^−^CD21^low^CD27^+^) [9–11]. Moreover, a relevant expansion of B cell subpopulations characterized by a CD21^low^CD27^−^ phenotype has been recently described in chronically infected patients in advanced stage of HIV-1 infection with a pronounced CD4^+^ T cell lymphopenia and ongoing HIV-1 replication [12]. This B cell population, which is normally present at low frequency in the peripheral blood of healthy individuals, can be further divided, depending on the expression of CD10, into immature/transitional B cells (CD10^+^) or exhausted tissue-like memory B cells (CD10^−^) [13–15].

Torque teno virus (TTV) is a single-strand DNA virus harmlessly carried by the majority of healthy individuals [16]. TTV is considered a surrogate marker of the immunological status of the host [17]. In fact, TTV viral load has been found significantly higher in HIV-1-infected patients presenting AIDS and decreased survival than healthy individuals [18].

Here we demonstrate that asymptomatic patients naïve for HAART with preserved CD4^+^ T cell counts (>350 cells/*μ*L) show an aberrant B cell subsets distribution and a concurrent increase in TTV viral load compared to HIV-1-uninfected individuals as well as HAART successfully treated patients.

## 2. Materials and Methods

### 2.1. Study Population

Blood samples were obtained from a cohort of 43 chronic HIV-1-infected patients naïve for HAART and without any history of opportunistic infections or malignancies. Clinical features of these patients are described in Table 1. Blood samples were also obtained from a cohort of 20 HAART-treated patients, aviremic for at least 12 months (Table 2), and from 34 healthy laboratory workers. The HIV-1-infected patients enrolled in this study were recruited at the Institute of Infectious and Tropical Diseases of the University of Brescia, provided their written informed consent. The study was approved by the Ethical Committee of the University Hospital of Brescia according to the declaration of Helsinki.

### 2.2. FACS Analysis

Blood samples were analyzed for 3- or 4-colors surface staining after lysis of red blood cells (FACS Lysing Solution, BD Bioscences, Milan, Italy). Monoclonal antibodies directly conjugated either to fluorescein-isothiocyanate (FITC), or phycoerythrin (PE), or peridinin-chlorophyll-protein (PerCP), or AlloPhycocyAnin (APC) specific for CD10, CD19, CD21, CD27, CD3, CD4, CD8, and CD127 were used (BD Bioscences). Flow cytometric analysis was performed using FACSCalibur flow cytometer (BD Biosciences), and 50.000 events (gated on lymphocytes) were acquired. Data were analyzed by CellQuest software.

### 2.3. IL-7 Serum Levels Detection

Detection of IL-7 serum concentration was performed through Quantikine HS IL-7 Immunoassay, according to the protocol provided by the supplier (R&D Systems, Minneapolis, USA).

### 2.4. Virus Detection and Quantification

HIV-1 plasma viral load was detected using Roche Amplicor PCR kit (RocheDiagnostic, Milan, Italy). DNA extracted from 200 *μ*L of plasma samples was examined for TTV genome using a single step universal TaqMan real-time PCR assay as previously described [17]. The sensitivity of the assay was of 3.0 log_10_ DNA copies/mL of plasma. All samples were tested simultaneously in triplicate.

### 2.5. Statistical Analysis

Statistical analysis were performed using GraphPad Prism v4.0c for Mac OS X. The distributions of each immune parameter between healthy donors and HIV-1-infected patient cohorts were compared using the nonparametric Mann-Whitney *U* test. All *P* values were 2 sided and unadjusted. Correlation between variables was tested using Spearman's rank correlation coefficient test. For all statistical analysis, the 0.05 level of significance was used (*P* < 0.05). Data in the text were expressed as median.

## 3. Results

### 3.1. Several B Cell Subset Alterations in Asymptomatic and HAART-Naïve HIV-1-Infected Patients

In the present study we examined the frequency and the function of B cell populations in 43 HIV-1-infected patients characterized by being clinically asymptomatic, with CD4^+^ T cell count >350 cell/*μ*L (median: 510 cell/*μ*L), median viral load of 6779 RNA copies/mL and HAART-naïve. As control, B cell populations were examined in 34 healthy individuals.

**Figure 1:**
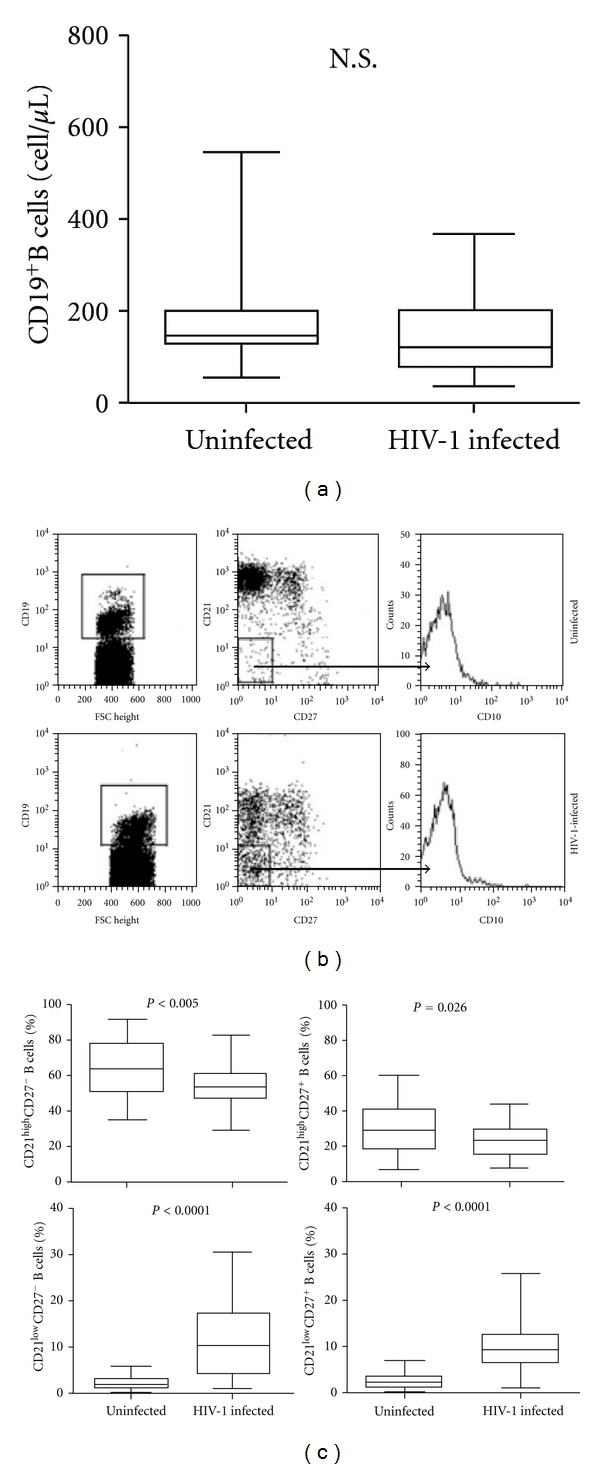
Peripheral blood B cell subsets alterations in asymptomatic HIV-1-infected patients. (a) Comparative analysis of CD19^+^ B cell counts (cell number/*μ*L) in HIV-1-uninfected individuals and asymptomatic HIV-1-infected patients. (b) Analysis of CD21 and CD27 expression on B cells from HIV-1-uninfected individuals and asymptomatic HIV-1-infected patients, and analysis of CD10 expression on CD21^low^/CD27^−^ B cells. Percentages of B cell subsets were determined by four-color flow cytometry analysis of CD19, CD21, CD27, and CD10 surface expression molecules. Profiles of expression of CD21 and CD27 are shown for one representative of each group. Cells were gated according to the lymphocytes forward and side scatter pattern and the CD19^+^ cells. (c) Comparative analysis of B cell subpopulations in HIV-1-uninfected individuals and asymptomatic HIV-1-infected patients. Frequency of CD21^high^CD27^−^ naïve B cells (upper left panel), CD21^high^CD27^+^ resting memory B cells (upper right panel), CD10^−^CD21^low^CD27^−^ exhausted tissue-like memory B cells (lower left panel), and CD21^low^CD27^+^ mature/activated B cells (lower right panel) are shown for the total of donors. Differences between groups were evaluated using the two-tailed Mann-Whitney *U* test, and were considered significant at *P* < 0.05. Into the box plots, horizontal lines represent median values for each group, boxes show the 25th and 75th percentiles, and bars show SD. N.S. = not significant.

**Figure 2:**
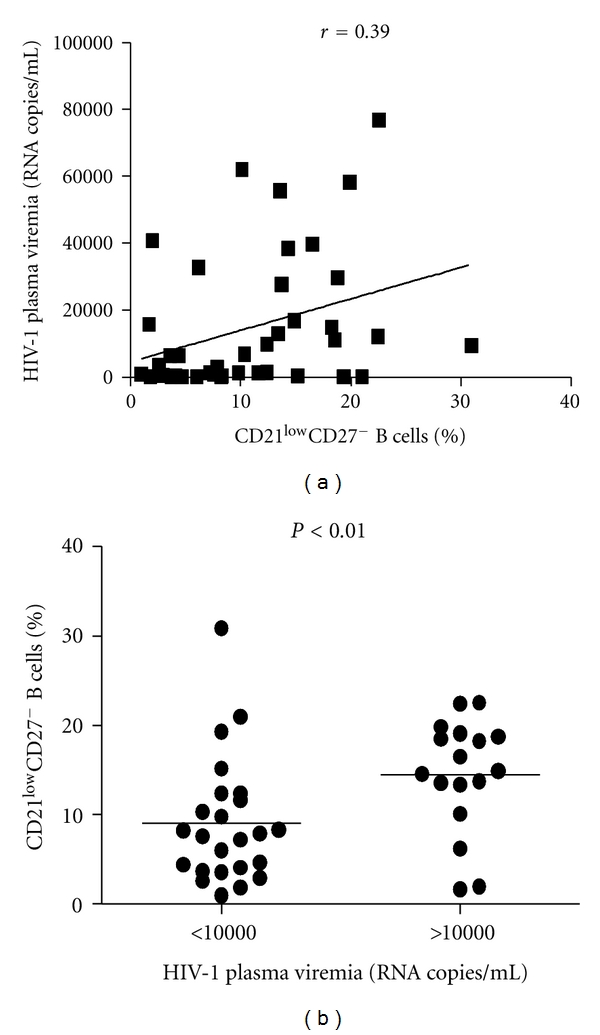
Association between exhausted tissue-like memory B cells and HIV plasma viremia. Correlation between CD10^−^CD21^low^CD27^−^ B cell percentages and HIV-1 plasma viremia (RNA copies/mL) in asymptomatic HIV-1-infected patients (a). Comparative analysis of CD10^−^CD21^low^CD27^−^ B cell percentages in asymptomatic patients with HIV-1 plasma viremia ≤ to 10000 RNA copies/mL and asymptomatic patients with HIV-1 plasma viremia > to 10000 RNA copies/mL (b). Association of 2 different data groups were analyzed by Spearman's rank test; *P* and rho values are indicated. Each symbol represents one individual, and regression line is shown. Differences between groups were evaluated using the two-tailed Mann-Whitney *U* test, and were considered significant at *P* < 0.05. Into the box plots, horizontal lines represent median values for each group, boxes show the 25th and 75th percentiles, and bars show SD.

We observed that the median of CD19^+^ B cell counts of the asymptomatic HIV-1-infected patients was superimposable to the median of healthy individuals (129.5 versus 143 cell/*μ*L, resp.) (Figure 1(a)). Moreover, the frequency of B cell subsets, such as mature/activated, immature/transitional or tissue-like memory, resting/memory, and naïve B cells was analyzed in all tested individuals. Although the normal number of total B cells, several perturbations were detected in the B cell subpopulations of asymptomatic HIV-1-infected patients. As shown in Figures 1(b) and 1(c) among the CD21^high^ B cells we observed that the frequencies of both resting/memory (CD27^+^), and naïve (CD27^−^) were significantly lower in the peripheral blood of the asymptomatic HIV-1-infected patients compared to healthy donors (median percentage: 23.5 versus 29.4, *P* = 0.026, and 54.2 versus 64.4, *P* < 0,005, resp.). However, we observed that activated/mature CD21^low^CD27^+^ and immature/transitional or tissue-like memory CD21^low^CD27^−^ B cells were significantly higher in the asymptomatic HIV-1-infected patients compared to uninfected donors (median percentage: 9.6 versus 2.4, *P* < 0.0001, and 10.4 versus 2.1, *P* < 0.0001, resp.). A previous study from Malaspina et al. [13] showed that the frequency of CD21^low^CD27^−^ B cells was increased in HIV-1-infected patients in advanced stage of disease (CD4^+^T cell count <200 cell/*μ*L). This cell subset included both CD10^+^elements, accounting for an immature/transitional B cell phenotype, and CD10^−^ cells defining exhausted tissue-like memory B cells. In our cohort of asymptomatic HIV-1-infected patients the percentage of CD10^+^ B cells was particularly low and similar to those of healthy donors. The CD21^low^CD27^−^ B cells subtype was essentially negative for CD10, attesting for an exhausted tissue-like memory B cells phenotype (Figure 1(b)). The percentage of CD21^low^CD27^−^ B cells positively correlated to the HIV-1 viral load (*r* = 0.39, *P* = 0.012). A higher percentage of CD21^low^CD27^−^ B cells was detected in patients with high plasma viremia (>10000 RNA copies/mL), and, vice versa, a lower percentage of circulating CD21^low^CD27^−^ B cells was found in patients with low plasma viremia (<10000 RNA copies/mL) (*P* < 0.01) (Figure 2). No correlation was observed between the percentage of CD21^low^CD27^−^ B cells and CD4^+^ T cell count (*r* = −0.27, *P* = 0.085, not shown).

### 3.2. Increased Levels of IL-7 in Sera of Asymptomatic HIV-1-Infected Patients

**Figure 3:**
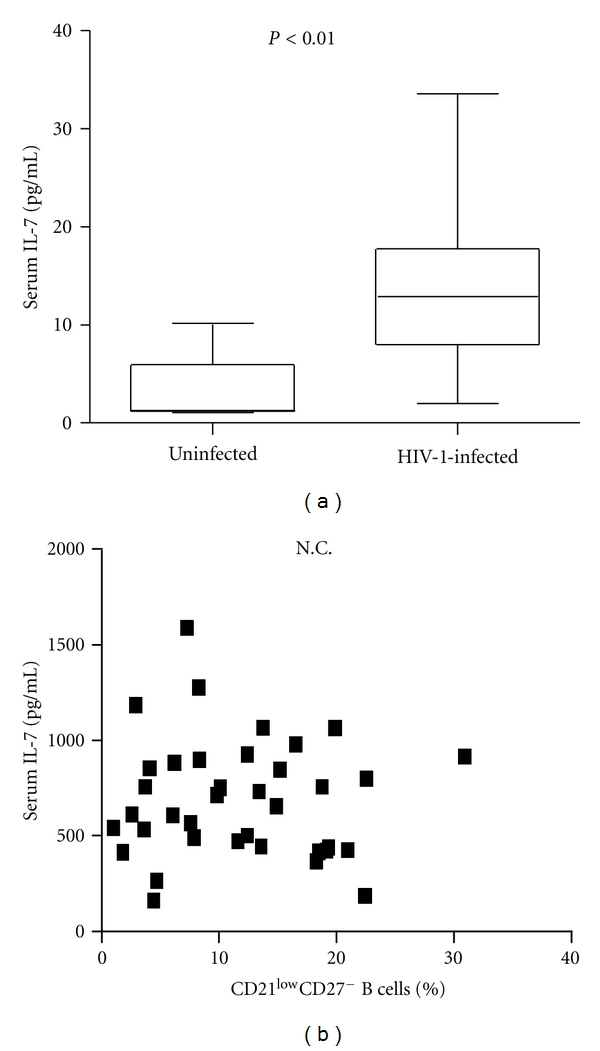
IL-7 serum levels in asymptomatic HIV-1-infected patients and correlation with CD10^−^CD21^low^CD27^−^B cells frequency. (a) Comparative analysis of IL-7 serum levels (pg/mL) in HIV-1-uninfected donors and asymptomatic HIV-1-infected patients. ELISA was performed to measure the IL-7 concentration. (b) Correlation between IL-7 serum levels and percentage of CD10^−^CD21^low^CD27^−^ B cells in asymptomatic HIV-1-infected patients. Differences between groups were evaluated using the two tailed Mann-Whitney *U* test, and were considered significant at *P* < 0.05. Into the box plots, horizontal lines represent median values for each group, boxes show the 25th and 75th percentiles, and bars show SD. Association of 2 different data groups were analyzed by Spearman's rank test. Each symbol represents one individual, and regression line is shown. N.C.= no correlation.

IL-7 is a pleiotropic cytokine controlling T lymphopoiesis and T cell peripheral expansion through interaction with its surface receptor (IL-7R*α* or CD127) [19]. Several investigators observed increased serum levels of IL-7 in HIV-1-infected patients characterized by severe CD4^+^ lymphopenia, and showed direct correlation between IL-7 serum concentration and viral load, loss of CD4^+^ T cells, and alteration of B cell subsets [13, 20]. Moreover, the frequency of immature/transitional CD10^+^CD21^low^CD27^−^ B cells is positively correlated to the IL-7 concentration detected in lymphopenic HIV-1-infected patients [13]. For these reasons, high levels of IL-7 are considered a hallmark of advanced HIV-1 disease. However, we observed a dramatic increase of IL-7 serum levels also in asymptomatic HIV-1-infected patients compared to healthy individuals (median concentration: 12.9 pg/mL versus 1.7 pg/mL, *P* < 0.01) despite a relatively preserved number of CD4^+^ T cells (median number count: 510 versus 831 cell/*μ*L) (Figure 3(a)). No correlation between IL-7 serum levels and CD4^+^ T cell counts or exhausted tissue-like memory B cell subset frequency were detected (Figure 3(b)).

### 3.3. Increase of TTV Load in Asymptomatic HIV-1-Infected Patients and Correlation with the CD21^low^CD27^−^ B Cells Frequency

So far, our findings showed profound phenotypic alterations in the B cell compartment of patients in the asymptomatic stage of HIV-1 infection. Previous studies showed that B cell subsets, abnormally expressed in the peripheral blood of HIV-1-infected patients in advanced stages of disease, and scarcely detectable in healthy individuals, displayed several functional defects [13, 21]. The aim of our study was trying to understand the impact of these uncommon B cell subsets on immunological surveillance *in vivo*.

**Figure 4:**
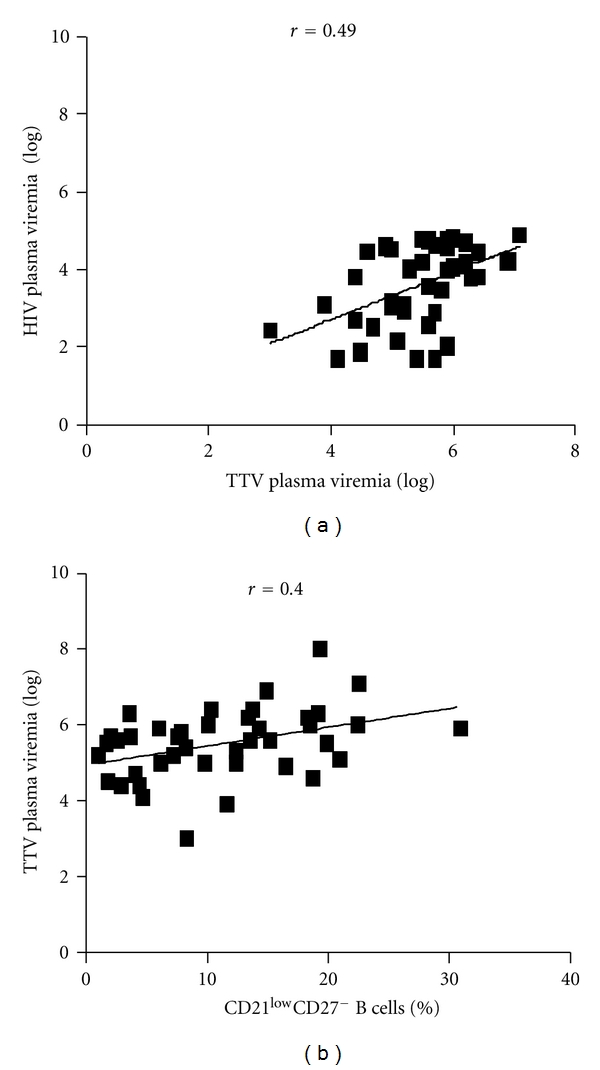
Perturbation in TTV immune surveillance in asymptomatic HIV-1-infected patients. (a) Correlation between TTV and HIV-1 viral loads (log⁡ DNA copies/mL, and log⁡ RNA copies/mL, resp.) in plasma derived from asymptomatic HIV-1-infected patients. TTV and HIV viral loads were measured by PCR and RT-PCR, respectively. (b) Correlation between plasma TTV titer and percentage of CD10^−^CD21^low^CD27^−^ B cells in asymptomatic HIV-1-infected patients. Association of 2 different data groups were analyzed by Spearman's rank test; *P* and rho values are indicated. Each symbol represents one individual, and regression line is shown.

TTV is an ubiquitous virus which infects up to 80% of the healthy population [22], and that is present at high titer in the blood of patients in advanced stages of HIV-1 infection compared to healthy subjects. In HIV-1-infected patients TTV load inversely correlated with the CD4^+^ T cell count [18]. TTV plasma viremia was measured in all individuals enrolled in our study. Asymptomatic HIV-1-infected patients showed a significantly higher amount of circulating TTV compared to healthy individuals (titer median: 5.7 versus 4.6 log⁡ DNA copies/mL, *P* < 0.005, Table 3). A direct correlation was observed between TTV load and both HIV-1 viremia and percentage of CD21^low^CD27^−^ B lymphocytes (*r* = 0.49  *P* < 0.001, and *r* = 0.40, *P* < 0.001 resp.) (Figure 4). These data suggest that in asymptomatic HIV-1-infected patients the increased percentage of CD21^low^CD27^−^ B cells may be related to the lack of *in vivo *immunological control of TTV replication.

### 3.4. Normal B Cell Subsets Frequency and Normal TTV Plasma Levels in HAART-Treated Patients

**Figure 5:**
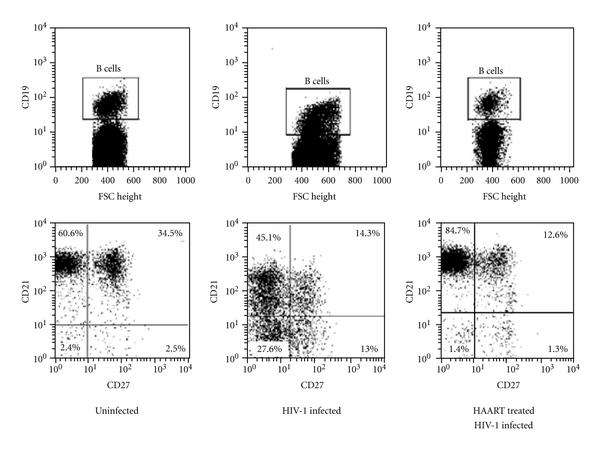
Effect of HAART in B cell subsets distribution in HIV-1-infected patients. Comparative analysis of CD21 and CD27 expression on B cells from HIV-1-uninfected individuals, asymptomatic HIV-1-infected patients, and HAART-treated patients. Profiles of expression of CD21 and CD27 are shown for one representative of each groups (lower panel). Cells were gated according to the lymphocytes forward and side scatter pattern and the CD19^+^ cells (upper panel).

We then investigated the effect of HAART on the B cell compartment of a cohort of HIV-1-infected individuals who experienced low levels of CD4^+^ T cell counts during the course of infection (median nadir: 183 cell/*μ*L), and maintained undetectable HIV-1 viral load for the last 12 months of therapy. At the time of our study, all patients belonging to this cohort displayed CD4^+^ T cell counts higher than asymptomatic therapy-naïve HIV-1-infected patients (median 682 cells/*μ*L versus 510 cell/*μ*L, *P* < 0.0001). The absolute number of B cells was significantly higher in HAART-treated patients as compared to both asymptomatic HIV-1-infected HAART-naïve patients and healthy individuals (median: 233 cell/*μ*L versus 129.5 cell/*μ*L, *P* < 0.005, and versus 143 cell/*μ*L, *P* < 0.01, resp.) (Table 3). In the cohort of HAART-treated patients we observed no presence of uncommon B cell populations with percentages of CD21^low^CD27^−^ and of CD21^low^CD27^+^ B cells superimposable to those of healthy individuals (Figure 5). At the same time, the percentage of CD21^high^CD27^−^ B cell subpopulation was higher in HAART-treated than asymptomatic HIV-1-infected cohort patients (*P* < 0.0005), and the IL-7 levels of treated-patients were superimposable to those of healthy donors. Finally, a successful immunological surveillance was observed in HAART-treated patients as they displayed a TTV plasma viremia within ranges commonly observed in healthy subjects (median: 4.1 versus 4.6 log⁡ DNA copies/mL).

## 4. Discussion

Disturbances in differentiation and function of B cells characterize HIV-1 infection, and mostly, these impairments are correlated to the loss of CD4^+^ T cells and the increase of HIV-1 load [12]. In the present study, we demonstrate that asymptomatic HIV-1-infected patients naïve for HAART are characterized by normal B cell numbers but impaired B cell subsets frequencies. In particular, we observed that CD21^low^CD27^−^ and CD21^low^CD27^+^ B cells were overexpressed, whereas the frequencies of CD21^high^CD27^−^ and CD21^high^CD27^+^ B cells, which respectively account for naïve and resting memory B cells, were lower compared to healthy donors. The increased uncommon CD21^low^CD27^−^ B cell population in the asymptomatic HIV-1-infected patients consisted exclusively of exhausted tissue-like memory B cells since no CD10 molecule surface was detected in this cellular subset. It is likely that the appearance of CD21^low^CD27^+^ B cells, which have undergone HIV-1-induced activation and differentiation to plasma blasts, and are usually referred as activated mature elements, as well as the appearance of CD21^low^CD27^−^ B cells, which derive from exhausted B cell subpopulation, can be ascribed to the B cell hyperactivation driven by the chronic yet asymptomatic presence of HIV-1 in patients. The presence of exhausted B cells in asymptomatic patients is strongly correlated to the HIV-1 load but not to the CD4^+^ T cell counts. Immature/transitional CD10^+^CD21^low^CD27^−^ B cells, previously described in the peripheral blood of HIV-1 positive patients in advanced stages of disease, were not present in the cohort of asymptomatic patients probably because they appear consequently to a strong reduction of CD4^+^ T cell counts [13, 15]. In fact, immature/transitional B cells were also observed in patients with non-HIV-1-related idiopathic CD4^+^ lymphopenia suggesting that HIV-1-induced CD4^+^ lymphopenia (and not HIV-1 viremia itself) drives the expansion of these elements in HIV-1-infected patients [23]. Therefore, our data show that exhausted tissue-like memory B cells emerge independently from immature/transitional B cells in HIV-1-infected patients, and precede the appearance of the latter possibly arising from homeostatic pressures induced by lymphopenia, and/or from conditions that increase the expression levels of early B cell stimulatory factors such as IL-7 [13, 23].

Several studies report that CD4^+^ lymphopenia driven by HIV-1 infection or bone marrow transplantation and idiopathic CD4^+^ T lymphopenia are often associated with increased serum levels of IL-7 [21, 23–25]. In order to explain the increased levels of IL-7 in CD4^+^ T lymphopenic individuals, it has been suggested that IL-7 augmentation occurs as part of a compensatory effect to enhance differentiation, survival, and expansion of T cells in order to respond to the HIV-1-mediated T cell depletion [24]. Surprisingly, we detected increased levels of IL-7 in sera of asymptomatic HIV-1-infected patients, but no correlations with CD4^+^ T cells count or HIV-1 load were detected. This result may find a possible explanation in the recent discovery by Guimond et al. [26] that systemic IL-7 concentrations increase solely because of diminished use of the lymphokine by target cells.

So, it is possible to hypothesize that the presence of exhausted memory B cells in asymptomatic HIV-1-infected patients is likely to prejudice the proper functioning of immune cells, and, therefore, a suitable immune surveillance.

So far, no pathological effect can be ascribable to TTV [22, 27]. TTV viral load seems to depend on the immunological status of the host, being patients with tumours or HIV-1 infection characterized by a higher TTV load compared to healthy individuals [16, 28]. Moreover, recent findings obtained by studying TTV in bone marrow transplanted patients show that the increase of viral loads is correlated with an increase in the percentages of dysfunctional CD8^+^CD57^+^ T lymphocytes circulating in blood [17, 29]. In the study we show that asymptomatic HIV-1-infected individuals have TTV viral load higher than healthy controls and that its titre is directly correlated to the HIV-1 viral load but not to the CD4^+^ T cells count. Moreover, TTV viral load directly correlated to the percentage of CD21^low^CD27^−^ B cells. These correlations may attest for a scarce immune control of TTV replication by B cells and for an already compromised immune surveillance in untreated asymptomatic HIV-1-infected patients.

Several studies have shown that HAART-mediated suppression of HIV-1 plasma viremia is followed by normalization of B cell counts and hyperactivation. In addition, reduction of HIV-1 viremia by HAART is associated with decreased frequency of exhausted tissue-like memory B cells [30]. Here we have described that patients who experienced HAART, leading to an undetectable HIV-1 viremia for the last 12 months of therapy, display a normal B cell subsets distribution. The role of HAART in the normalization of IL-7 serum levels is controversial: some investigators showed only a partial decrease in the IL-7 levels, never declining toward normal values whereas others documented a complete normalization of this immunological parameter [19, 31]. In our study, successfully HAART-treated patients showed IL-7 values superimposable to those of healthy HIV-1-uninfected individuals.

Interestingly, we reported that all HAART-treated patients experienced TTV load to levels usually detected in healthy individuals. This finding shows that antiretroviral therapies aimed at controlling HIV-1 load and normalizing B cell subpopulations can help to recover immune functions to levels capable of controlling the replication of this endogenous virus. Normalization of B cell counts and subpopulations by HAART is followed by improved B cell responses to both T cell-independent and T cell-dependent immunogens [29, 32–34]. Of particular interest is the recent observation that an early control of viral replication through HAART preserves the longevity of B cell responses in vaccinated HIV-1-infected children thus underscoring the direct role of HIV-1 viremia in B cell terminal differentiation [35]. These observations have important implications not only for preserving immune responses against secondary pathogens but also for maintaining or boosting HIV-1 specific immune responses, including antibody responses.

## 5. Conclusions

We have provided evidences that asymptomatic HIV-1-infected patients with a preserved CD4^+^ T cell number, and naïve for HAART, display alterations in B cell subset phenotype and impaired immune responses, as manifested by their inability to control TTV replication. On the other hand, normal B cell phenotypes and counts were found in patients who initiated HAART during chronic disease and displayed a negative HIV-1 viral load in the last 12 months of therapy. Normal B cell subpopulations in HAART-treated patients were mirrored by a normal TTV viral load, attesting for a recovery of adequate immunological surveillance. From this point of view, HAART as well as therapies aimed at decreasing immune cell deregulation can help to better control the loss of immune function if administered earlier during HIV-1 infection.

## Figures and Tables

**Table 1:** Profiles of asymptomatic HIV-1-infected patients.

Patients	Sex	Age (years)	HIV-1 risk group	CDC stage	HIV-1 viral load (RNA copies/mL)	CD4 T cell count (cell/*μ*L)
1	M	49	Heterosex	A1	6342	500
2	F	33	Heterosex	A2	760	471
3	M	30	Heterosex	A1	<50*	650
4	F	40	Heterosex	A1	9893	470
5	F	21	Heterosex	A1	160116	786
6	F	72	Heterosex	A1	2940	536
7	M	55	Homosex/bisex	A1	76871	477
8	M	27	Heterosex	A1	39880	540
9	F	44	Heterosex	A1	9337	487
10	F	42	Heterosex	A1	74	857
11	F	48	Heterosex	B1	62078	505
12	M	48	Unknown	A1	6778	384
13	M	28	Unknown	A1	58322	622
14	M	26	Homosex/bisex	A1	274979	550
15	F	36	Drugs user	B1	1332	454
16	M	43	Homosex/bisex	A2	27684	356
17	F	43	Heterosex	A1	1201	417
18	M	30	Drugs user	A1	15756	523
19	M	33	Heterosex	A1	789	742
20	M	42	Hematic product contact	A1	107	570
21	M	47	Drugs user	A1	323	552
22	M	47	Drugs user	A1	<50	684
23	M	50	Heterosex	A1	55681	510
24	M	55	Heterosex	A2	67431	481
25	M	45	Heterosex	A1	14781	386
26	F	46	Heterosex	A1	143	494
27	F	54	Heterosex	A1	276	417
28	M	32	Homosex/bisex	A1	11091	685
29	M	28	Heterosex	A1	362	620
30	M	34	Heterosex	A1	16841	668
31	F	48	Drugs user	A1	<50	640
32	M	44	Drugs user	A1	<50	2049
33	F	24	Drugs user	A2	49716	469
34	M	49	Homosex/bisex	A2	3569	489
35	F	28	Heterosex	A1	6390	555
36	M	49	Heterosex	A1	487	834
37	M	39	Heterosex	A1	32750	523
38	M	35	Homosex/bisex	A2	1121	408
39	M	49	Homosex/bisex	A1	13057	496
40	M	39	Homosex/bisex	A2	40811	478
41	M	40	Homosex/bisex	A2	1713	474
42	M	43	Homosex/bisex	A2	29763	355
43	M	43	Homosex/bisex	A1	62035	574

*<50 HIV-1 RNA copies/mL: undetectable levels.

**Table 2:** Profiles of HAART-treated HIV-1-infected patients.

Donors	Sex	Age (years)	HIV-1 risk group	HIV-1 viral load	CD4 T cell count (cell/*μ*L)	CDC before therapy	Nadir	Therapy
1	M	44	Drug user	<50	481	A3	93	EFV+FTC+TDF
2	M	43	Drug user	<50	662	A2	201	NVP+3TC+TDF
3	F	34	Heterosex	<50	1124	A2	308	NVP+AZT+3TC
4	M	36	Homosex/bisex	<50	1031	C3	9	NVP+FTC+TDF
5	M	46	Drug user	<50	740	C3	43	AZT/r+FTC+TDF
6	M	60	Heterosex	<50	512	C3	39	AZT+3TC+ABC
7	M	48	Heterosex	<50	731	A2	208	NVP+3TC+ABC
8	M	53	Heterosex	<50	566	A3	94	EFV+FTC+TDF
9	M	44	Drug user	<50	527	C2	274	AZT/r+3TC+TDF
10	F	41	Heterosex	<50	636	A2	311	EFV+3TC+ABC
11	M	56	Homosex/bisex	<50	638	C3	84	AZT/r+3TC+ABC
12	M	40	Drug user	<50	823	A2	365	AZT+3TC+ABC
13	M	49	Heterosex	<50	754	A3	23	AZT+3TC+ABC
14	F	31	Drug user	<50	633	A2	280	FSP/r+3TC+ABC
15	F	49	Drug user	<50	846	B3	57	NVP+FTC+TDF
16	M	39	Homosex/bisex	<50	701	A2	332	AZT/r+FTC+TDF
17	F	43	Heterosex	<50	1067	A2	339	EFV+FTC+TDF
18	F	40	Drug user	<50	524	B3	166	NVP+3TC+ABC
19	M	44	Homosex/bisex	<50	701	A2	239	NVP+FTC+TDF
20	M	44	Heterosex	<50	564	C3	23	LPV/r+FTC+TDF

<50 HIV-1 RNA copies/mL: undetectable table.

**Table 3:** Effects of HAART on B cells frequency, IL-7 serum levels, and TTV viral load.

	Healthy	Asymptomatic HIV-1 infected	HAART treated	P (HAART treated versus healthy)	P (HAART treated versus asymptomatic HIV-1 infected)
CD19^+^ B cell count (cell/*μ*L)	143.0	129.5	232.5	<0.01	<0.005
CD21^−^CD27^−^ B cell frequency (%)	2.1	10.4	2.7	NS	<0.0001
CD21^−^CD27^+^ B cell frequency (%)	2.4	9.6	1.7	NS	<0.0001
CD21^+^CD27^+^ B cell frequency (%)	29.4	23.5	22.4	<0.05	NS
CD21^+^CD27^−^ B cell frequency (%)	64.4	54.2	73.0	NS	<0.0005
IL-7 serum level (pg/mL)	1.7	12.9	2.0	NS	<0.01
TTV plasma viremia (log⁡ DNA copies/mL)	4.6	5.7	4.1	NS	<0.001

NS: not significant.

Results are expressed as median.
